# Glutathione: Pharmacological aspects and implications for clinical use in non-alcoholic fatty liver disease

**DOI:** 10.3389/fmed.2023.1124275

**Published:** 2023-03-22

**Authors:** Giovanni Santacroce, Antonella Gentile, Simone Soriano, Andrea Novelli, Marco Vincenzo Lenti, Antonio Di Sabatino

**Affiliations:** ^1^Department of Internal Medicine and Medical Therapeutics, University of Pavia, Pavia, Italy; ^2^First Department of Internal Medicine, San Matteo Hospital Foundation, Pavia, Italy; ^3^Department of Health Sciences, Clinical Pharmacology and Oncology Section, Università Degli Studi di Firenze, Firenze, Italy

**Keywords:** chronic liver disorder, metabolic syndrome, non-alcoholic fatty liver disease, oxidative stress, oral glutathione

## Abstract

Glutathione is a tripeptide synthesized at cytosolic level, that exists in cells in a reduced form (thiol-reduced-GSH-) and in an oxidized form (disulfide-oxidized). The antioxidant function of GSH has led to speculation about its therapeutic role in numerous chronic diseases characterized by altered redox balance and reduced GSH levels, including, for instance, neurodegenerative disorders, cancer, and chronic liver diseases. Among these latter, non-alcoholic fatty liver disease (NAFLD), characterized by lipid accumulation in hepatocytes, in the absence of alcohol abuse or other steatogenic factors, is one of the most prevalent. The umbrella term NAFLD includes the pure liver fat accumulation, the so-called hepatic steatosis or non-alcoholic fatty liver, and the progressive form with inflammation, also known as non-alcoholic steatohepatitis, which is related to the increase in oxidative stress and reactive oxygen species, eventually leading to liver fibrosis. Although the pathogenetic role of oxidative stress in these diseases is well established, there is still limited evidence on the therapeutic role of GSH in such conditions. Hence, the aim of this review is to depict the current molecular and pharmacological knowledge on glutathione, focusing on the available studies related to its therapeutic activity in NAFLD.

## 1. Introduction

Glutathione is a tripeptide found in many tissues at relatively high concentrations, namely 1–10 mM in cells, similarly to glucose, potassium, and cholesterol, with a critical role in several physiological processes, such as redox balance preservation, reduction of oxidative stress through detoxification from xenobiotic and endogenous compounds, and immune system modulation (1). The action of glutathione on oxidative stress has led to speculation on the possible therapeutic role of this molecule for several chronic diseases with altered redox balance.

Non-alcoholic fatty liver disease (NAFLD) is the most common liver disease worldwide, characterized by an excessive hepatic fat accumulation in the absence of alcoholic abuse, steatogenic medications or others concomitant liver diseases (2). NAFLD could be considered an hepatic manifestation of metabolic syndrome, with a strong association with obesity, type 2 diabetes, hypertension, and dyslipidaemia (3). Given the close association between NAFLD and metabolic dysfunction, a panel of experts proposed in 2020, not without some controversy, to rename this condition as metabolic-(dysfunction) associated fatty liver disease (4).

Although the pathogenic role of oxidative stress in NAFLD pathogenesis is well established (5), there are limited studies available in the literature investigating the potential effect of glutathione supplementation in this condition. The aim of this narrative review is to provide a broad overview on the pharmacologic aspects of glutathione, with a focus on current clinical data on its use for metabolic liver disease.

## 2. Glutathione: Pharmacological aspects

Glutathione is a tripeptide (γ-L-glutamyl-L-cysteinylglycine) consisting of glutamate, cysteine and glycine, with an atypical peptide bond between glutamate residue and cysteine, *via* the γ-carboxyl group. It exists in cells in two states: thiol-reduced (GSH) and disulfide-oxidized (GSSG). The reduced form (GSH) is the predominant one, accounting for more than 98% of total glutathione. Most of the GSH (80–85%) is stored in the cytosol, 10–15% in the mitochondria (with an equal concentration between matrix and cytosol, thus requiring specific transport systems) and a small part in the endoplasmic reticulum (ER) (1, 6, 7).

### 2.1. Thiol-reduced glutathione (GSH) synthesis

**Figure 1:**
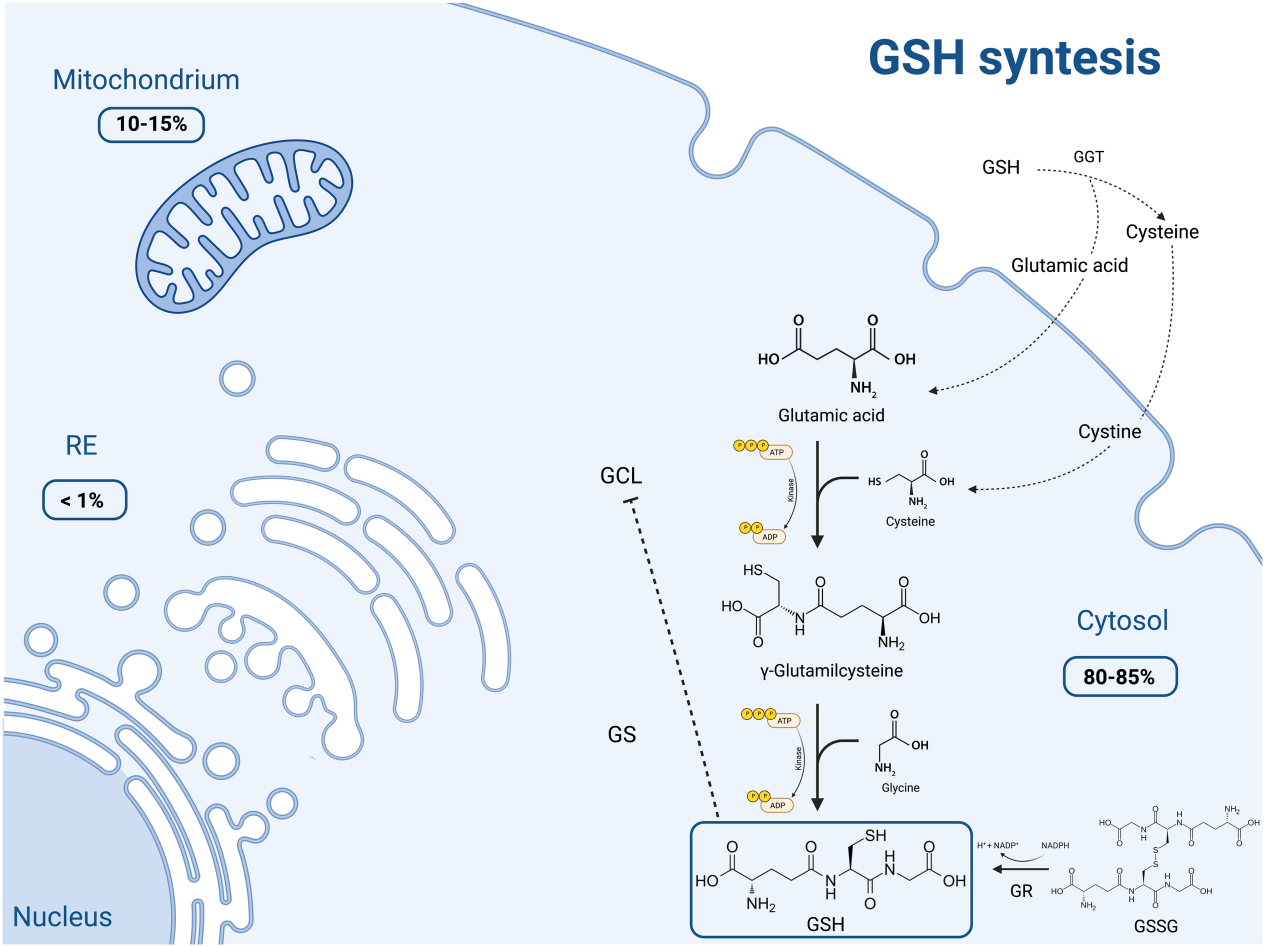
Schematic representation of the main molecular mechanisms of reduced glutathione (GSH) synthesis and the intracellular distribution of this molecule. GSH production occurs through three main pathways: (1) *de novo* synthesis *via* a 2-step process catalyzed by glutamate cysteine ligase (GCL) and glutathione synthetase (GS), which is primarily controlled by the cellular levels of cysteine. Moreover GCL activity is in part regulated by GSH feedback inhibition; (2) recycling of cysteine from conjugated glutathione *via* γ-glutamyltranspeptidase (GGT) in the γ-glutamyl cycle; (3) regeneration of the oxidized glutathione (GSSG) to GSH by glutathione reductase (GR). Most (80–85%) of the cellular GSH is in the cytosol; 10–15% is in the mitochondria and a small percentage is in the endoplasmic reticulum (ER). Created with “Biorender.com.” ADP, adenosine diphosphate; ATP, adenosine triphosphate; ER, endoplasmic reticulum; GCL, glutamate cysteine ligase; GGT, γ-glutamyltranspeptidase; GR, glutathione reductase; GS, glutathione synthetase; GSH, thiol-reduced glutathione; GSSG, disulfide-oxidized glutathione; NADP+, oxidized nicotinamide adenine dinucleotide phosphate; NADPH, reduced nicotinamide adenine dinucleotide phosphate; −--|, inhibition.

GSH is made available in cells through 3 processes, summarized in Figure 1.

The first process consists in the *de novo* synthesis of GSH from its component amino acids, through two ATP-consuming enzymatic reactions. It occurs exclusively in the cytosol, where glutamate cysteine ligase (GCL) and glutathione synthetase (GS) perform their function. The first step involves the binding of glutamic acid and cysteine, catalyzed by GCL, to form glutamylcysteine. This rate limiting step is controlled by the cellular availability of cysteine and GCL activity. This assumption is supported by the finding that only the overexpression of GCL, and not of GS, results in increased GSH levels (6). On the other hand, GSH exerts a negative feedback inhibition on GCL. In the second step, the homodimeric enzyme GS, a member of the ATP-grasp superfamily, rapidly catalyzes the binding of γ-glutamylcysteine with glycine, obtaining GSH (8, 9). GS, differently from GCL, is not feedback-inhibited by GSH and it is not associated with a regulatory subunit. Thus, GS activity appears to be mainly controlled by substrate availability.

The second pathway of GSH synthesis is that related to the recycling of cysteine. GSH is exclusively degraded at the extracellular level by cells expressing γ-glutamyltranspeptidase (GGT), such as those of the hepatobiliary tree and of other organs like the heart, kidney, lungs, pancreas, and seminal vesicles. GGT allows the degradation of GSH and the recycling of its constituent amino acids, such as glutamic acid and cysteine, to generate new GSH (so called γ-glutamyl cycle). The resulting cysteine is unstable at extracellular level, and it is rapidly autoxidizes to cystine. Nevertheless, cystine is taken up by some cells (i.e., endothelial cells) and, given the high reducing conditions, is intracellularly reduced to cysteine, employed for the synthesis of GSH (10, 11). This direct transport of extracellular cystine does not take place in hepatocytes, where the reduction of cystine to cysteine occurs mainly in the outer cell membrane as a consequence of GSH efflux (11). The extracellular L-cysteine/L-cystine redox balance, and thus the synthesis of GSH, is finely regulated by the intracellular conversion of L-cystine into L-cysteine (10), and its impairment is related to oxidative stress and other pathological disorders.

The last GSH synthetic process is the one depending on the conversion of the oxidized dimer GSSG to 2 reduced GSH molecules in cells by glutathione reductase (GR), an ubiquitous enzyme of the family of disulfide reductases, in the presence of NADPH and flavin adenine dinucleotide -FAD-. GR can perform its enzymatic activity in the cytoplasm, but also in the ER and within lysosomes, mitochondria and the nucleus. Since it participates in the synthesis of GSH, this enzyme plays a key role in the cellular redox homoeostasis (12).

The majority of plasmatic GSH originates from the liver, and for this reason an impairment in hepatic GSH synthesis has a systemic impact on redox balance and oxidative stress (1, 6, 7, 11).

### 2.2. The antioxidant role of GSH

GSH is implicated in several functions, including antioxidant defense with reduction of oxidative stress and maintenance of redox balance, metabolic detoxification from xenobiotics and exogenous compounds, cell cycle regulation, and immune system modulation, as well as fibrogenesis (1).

Its main role is to shield cellular macromolecules from endogenous and exogenous reactive oxygen species (ROS) and nitrogen ones. In particular, GSH catalytically detoxifies from hydroperoxides, peroxynitrite, and lipid peroxides and directly scavenges various oxidant molecules, like superoxide anion, hydroxyl radical, nitric oxide, and carbon radicals. Furthermore, GSH deals directly with heavy metals and persistent organic pollutants (POPs), direct causes of oxidative stress. POPs are mainly excreted through conjugation with GSH and this mechanism is extremely important for the health status, since exposure to POPs has been associated to diabetes, cardiovascular diseases and many other chronic diseases (1, 13).

ROS production can occur at several intracellular sites but, for most cells, takes place in the mitochondria and the mitochondrial electron transport chain is the main cellular process of ROS generation in physiological circumstances (14). For superoxide anion, the main ROS, a first line of defense is represented by the enzyme superoxide dismutase, localized in the mitochondrial matrix. This enzyme is able to convert the superoxide anion into hydrogen peroxide (H_2_O_2_). Once obtained, H_2_O_2_ can be degraded in mitochondria *via* the GSH redox system, employing glutathione peroxidases (Gpxs) and GRs, but also through peroxiredoxins (Prxs), a family of thiol-specific peroxidases.

Gpxs exist in multiple isoforms, with different cellular localization and different substrate specificity (15). Of these, Gpx1, localized mainly at the mitochondrial level (16), is the isoform most active in the liver (17). Gpxs are the main enzymes involved in scavenging ROS at high intracellular concentrations, protecting cells from oxidative stress-induced damage. At nanomolar concentrations of H_2_O_2_, Prxs seem to be more active, given their higher intracellular concentration and rate constant (18).

GSH, exploiting the redox-active thiol residue (-SH) of cysteine, exerts its antioxidant function mainly *via* Gpxs-mediated reactions, which result in peroxide buffering with simultaneous oxidation of GSH to GSSG (19). The obtained GSSG is potentially toxic and, under oxidative stress, its excessive accumulation can manifest its toxicity. First of all, GSSG may activate the SAPK/MAPK pathway, leading to cell apoptosis. In addition, GSSG retained in mitochondria during oxidative stress can lead to the S-gluthionylation of target proteins with mitochondrial dysfunction (20, 21). The S-glutathionylation is a process in which the interaction between GSSG and cysteinyl residues of proteins results in the formation of mixed disulfides (22). This physiological mechanism, which is useful for the post-translational modification of multiple proteins and for the regulation of signal and metabolic pathways, can turn harmful in case of oxidative stress, with the above-mentioned mitochondrial damage. To prevent the GSSG toxicity, this molecule is rapidly transformed in its reduced variant (GSH) by high intracellular levels of GRs, with the aim of maintaining an appropriate redox balance in the cell (1). Hence, the GSH/GSSG ratio represents a marker of oxidative stress.

Besides the neutralization of free radicals produced in phase 1 liver metabolism of chemical toxins, GSH also participates in the protection from the resulting electrophilic substrates through the intervention of glutathione-S-transferases (GSTs). GSTs are phase 2 enzymes, ubiquitously distributed in the cell. The ones locate in mitochondria have both GSH-transferase and peroxidase activity (23). They are therefore able, by exploiting the properties of GSH, to activate conjugation and peroxide reduction of dangerous products.

Also, GSH facilitates the transport and excretion of toxins, through the formation of S-conjugates of activated intermediates, which are water soluble and undergo renal excretion.

Finally, GSH is also a cofactor for several antioxidant enzymes. Among the antioxidant molecules of low molecular weight are vitamins E and C, obtained from the diet. In particular, vitamin E, after acting as an antioxidant by reducing lipid radicals, is restored to its reduced form by vitamin C. In turn, the oxidized vitamin C, thanks also to GSH, can revert to its reduced form (7). GSH therefore enables the recycling of vitamins C and E, again protecting the body from oxidative stress (1).

### 2.3. Depletion of GSH and therapeutic implications

The depletion of GSH levels has been demonstrated in aging and multiple chronic degenerative diseases, including neurodegenerative, cardiovascular, pulmonary, immune disorders and cancers (24, 25). There are cumulating data on reduced GSH levels and the consequent increased susceptibility to oxidative stress in many human diseases, contributing to the onset and worsening of these conditions.

For this reason, many studies have been conducted on the best methods to increase intracellular and intramitochondrial levels of GSH. A first approach to promote glutathione production might be the administration of specific precursors, cofactors or specific foods and nutrients that may increase or maintain optimal glutathione levels. Examples are cysteine supplements in the form of whey or *N*-acetylcysteine, antioxidant vitamins (B,C,E), alpha-lipoic acid, selenium or phytonutrients (i.e., Brassica vegetables and green tea) (25). However, data are scant and controversial, resulting in limited efforts to study the effect of nutritional interventions on GSH status. Further studies are needed to clarify optimal dose and delivery forms and one mandatory target should be the identification of sub-groups of individuals most likely to respond to particular supplements, nutrients or foods.

On the other hand, the obvious strategy to increase GSH levels is its direct administration. The main routes of administration of glutathione are oral, intramuscular, and intravenous. Intravenous GSH has a short half-life but has shown to be effective in several diseases. For example, the GSH intravenous administration in patients with Parkinson’s disease determined significant improvements, which lasted for 2–4 months after the administration (26). Also oral administration, although with conflicting results, resulted in increased serum GSH levels with reduced oxidative stress and beneficial effects in several diseases (27, 28). Richie et al. recently found that oral GSH at either 250 or 1,000 mg/day was associated to significant increase in the body storage of GSH in non-smoking adults, in a dose-dependent manner (27). They also noticed a decrease in the markers of oxidative stress at 6 months, as shown by the improvement in the GSSG/GSH ratio. Furthermore, recent studies suggested that GSH oral administration in liposomal or sublingual forms may have a better bioavailability, with a favorable impact on systemic GSH levels (29, 30). For example, a novel GSH formulation bypassing the gastrointestinal digestion through an oral absorption, gave positive results in raising GSH blood concentration *in vitro* and *in vivo* (31). Moreover, this molecule showed a promising hepatoprotective function in a murine model of acute liver injury (32).

## 3. Non-alcoholic fatty liver disease: Pathogenesis and clinical features

NAFLD is the most common liver disease, characterized by an excessive hepatic fat accumulation in the absence of alcoholic abuse, steatogenic medications or others concomitant liver diseases (2). NAFLD could be considered an hepatic manifestation of metabolic syndrome, given the strong association with obesity, type 2 diabetes, hypertension and dyslipidaemia (3). This entity comprises NAFL and NASH, the latter being its progressive form and affecting about 10–20% of patients.

NAFLD appears to be more frequent in industrialized countries, its global prevalence is about 25% and varies across different geographical areas, being higher in Middle East and South America and lower in Africa (33). The overall prevalence of NASH is uncertain, as it relies on third-level referral centers with availability of liver biopsies, and is estimated between 1.5 and 6.45% (34).

**Figure 2:**
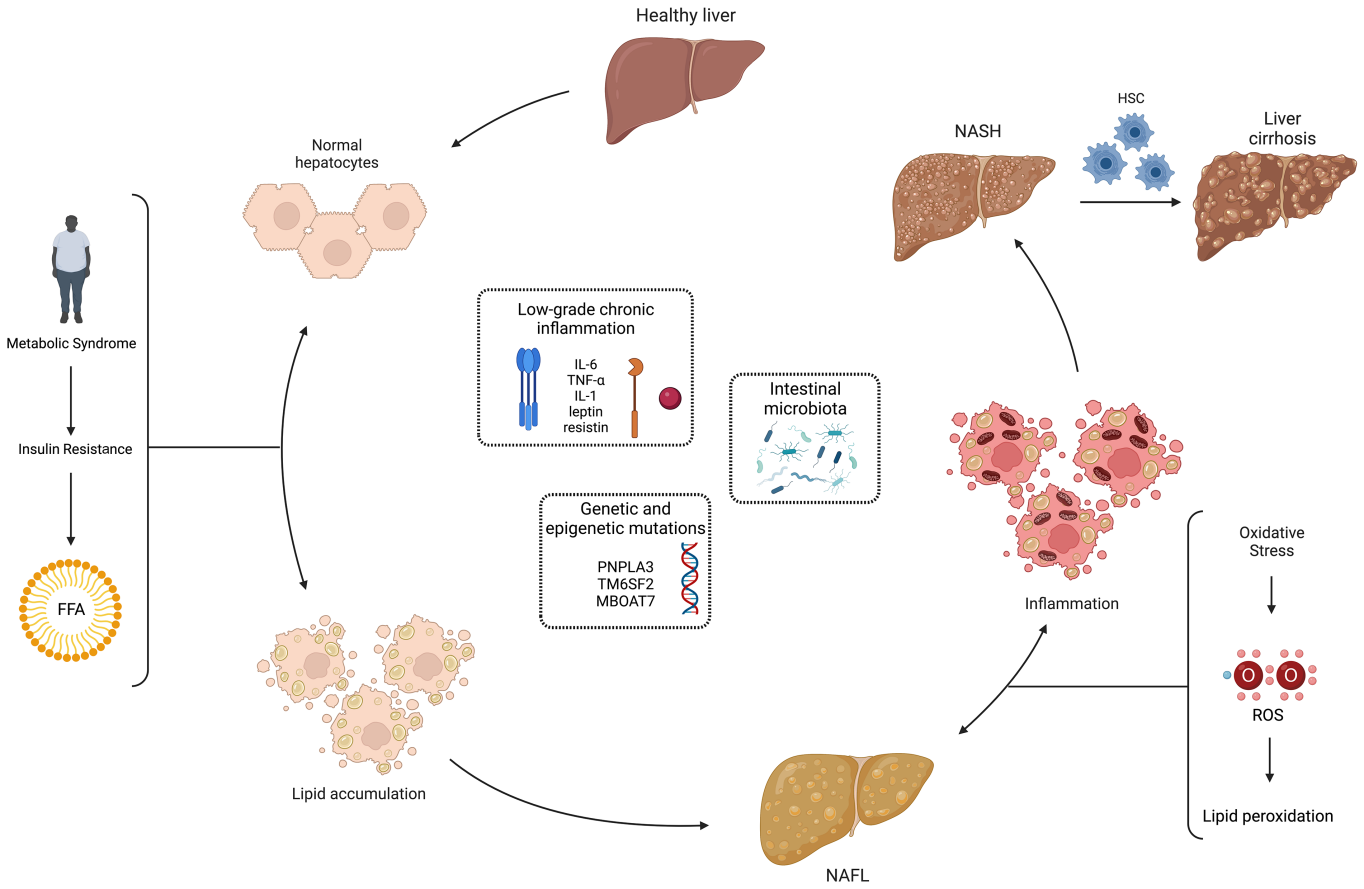
Non-alcoholic fatty liver disease (NAFLD) pathogenesis: “multiple hits” theory. The pathogenesis of NAFLD is multifactorial and multiple mechanisms have been proposed to explain the process of excessive liver lipid accumulation, with the subsequent possible inflammation and fibrosis. According to the classic “two-hits” model, the increased insulin resistance, secondary to metabolic syndrome, determines an excessive lipid accumulation in healthy hepatocytes (first “hit”), mainly through an increased mobilization of free fatty acids (FFA) from visceral adipose tissue to the liver. This process leads to the non-alcoholic fatty liver (NAFL), still reversible condition. The increased oxidative stress seems to be the second “hit” in the progression from NAFL to non-alcoholic steatohepatitis (NASH): the increase in reactive oxygen species (ROS) and consequently in lipid peroxidation has been associated to hepatocellular damage, inflammation and eventual fibrosis due to the activation of hepatic stellate cells (HSC). More recently, a “multiple hits” theory has been proposed, with the addition of other pathogenic factors in NAFLD development: low-grade chronic inflammation, genetic and epigenetic mutations, and intestinal microbiota. Created with “Biorender.com.” FFA, free fatty acids; HSC, hepatic stellate cells; IL, interleukin; MBOAT7, membrane bound O-acyltransferase domain containing 7; NAFL, non-alcoholic fatty liver; NASH, non-alcoholic steatohepatitis; PNPLA3, patatin-like phospholipase domain containing 3; ROS, reactive oxygen species; TM6SF2, transmembrane 6 superfamily member 2;TNF-α, tumor necrosis factor-alpha; ↔, reversible condition.

According to the European Clinical Guidelines, NAFLD is defined either by the presence of steatosis in >5% of hepatocytes at the liver biopsy or by a proton density fat fraction >5,6% assessed by proton magnetic resonance spectroscopy or magnetic resonance (2). NASH is defined by evidence of hepatocyte injury (ballooning) and inflammation, with or without fibrosis, in a liver biopsy. 20% of patients with NASH develop cirrhosis and have a high risk of hepatocarcinoma. (35). The pathogenesis of NAFLD, schematically represented in Figure 2, is multifactorial and multiple mechanisms have been proposed to explain the process of excessive liver lipid accumulation, with the subsequent possible inflammation and fibrosis (36). According to the classic “two-hits” model, the increased insulin resistance, secondary to metabolic syndrome, determines an excessive lipid accumulation in healthy hepatocytes (first “hit”), mainly through an increased mobilization of free fatty acids from visceral adipose tissue to the liver. This process leads to NAFL, still reversible condition. The increased oxidative stress seems to be the second “hit” in the progression from NAFL to NASH: the increase in ROS and consequently in lipid peroxidation has been associated to hepatocellular damage, inflammation, and eventual fibrosis due to the activation of hepatic stellate cells. Moreover, ROS inhibit hepatocyte secretion of VLDL and promote hepatic insulin resistance, inducing liver fat accumulation and necro-inflammation. The oxidative stress also contributes to atherosclerosis, representing a possible link between NAFLD and metabolic syndrome (37). In addition, antioxidants that protect the liver from ROS damage and lipid peroxidation may be depleted: GSH, vitamin E, vitamin C and beta-carotene were found to be reduced in the NASH setting (38, 39). Closely related to oxidative stress is the so-called dicarbonyl stress, i.e., the accumulation of dicarbonyl metabolites leading to cell and tissue disfunction (40). Altered functioning of the glyoxalase enzyme system, with associated accumulation of glycation products, has been shown as another possible pathogenic mechanism in NAFLD (41, 42).

More recently, a “multiple hits” theory has been proposed, with the addition of other pathogenic factors in NAFLD development: low-grade chronic inflammation, genetic and epigenetic mutations, and intestinal microbiota. A condition of low-grade inflammation has been related to NAFLD, with an abnormal production of cytokines and adipokines, such as interleukin (IL)-6, tumor necrosis factor (TNF)-α, IL-1, leptin. In particular, lower levels of adiponectin and increased expression of TNF-α and its soluble receptor have been recently related to the development of NASH (43). A role for genetic has been demonstrated, with evidence of an increased risk of NAFL in patients with polymorphisms, like those associated to patatin-like phospholipase domain containing 3 (PNPLA3) rs738409, transmembrane 6 superfamily member 2 (TM6SF2) rs58542926 and membrane bound O-acyltransferase domain containing 7 (MBOAT7) rs641738 (44–47). Conversely, patients who carry variants in hydroxysteroid 17-beta dehydrogenase 13 (HSD17B13, rs72613567) and mitochondrial amidoxime-reducing component 1 (MARC1, rs2642438) are more protected than the general population (48, 49). Finally, the gut microbiome may contribute to NAFLD pathogenesis: an increased intestinal permeability could lead to the entry of endotoxins in portal circulation and activation, through toll-like receptor 4, of Kupffer cells, with consequent inflammation (50–53).

Most patients with NAFLD are asymptomatic, and the diagnosis is mainly made incidentally on the basis of liver biochemistry or abdominal ultrasound abnormalities. Common symptoms include right upper quadrant pain and fatigue. The most common laboratory alterations are elevation of liver enzymes, with serum alanine aminotransferase higher than aspartate aminotransferase (ALT>AST) (54), although transaminase levels may be within limits in more than one third of cases, and hyperferritinemia (55), which has been demonstrated to be a marker of sever histologic damage and an independent predictor for liver fibrosis (56, 57). In a recent work by Corradini et al., variants of genes related to iron metabolism were shown to be associated with hyperferritinemia and more severe NAFLD (58).

Diagnosis is made by exclusion of alcohol abuse and other causes of liver disease (HBV-related hepatitis, HCV-related hepatitis, autoimmune liver diseases, polycystic ovary syndrome, drug-induced liver disease and congenital causes such as hereditary hemochromatosis, Wilson’s disease, alpha-1 antitrypsin deficit). In association with the assessment of liver enzymes levels in serum, ultrasound (US) is the first line procedure to screen patients for NAFLD. Although US in a non-invasive and practical method, it has low sensitivity for mild levels of steatosis and cannot be used for the distinction between NAFLD and NASH, without a concomitant liver biopsy. Thus, vibration-controlled transient elastography (VCTE) or magnetic resonance elastography are used to identify early phases of the disease. If significant fibrosis is confirmed, patients should be referred to a specialist to perform liver biopsy and confirm the diagnosis histologically (59). In Chinese guidelines, high serum levels of CK-18 fragments (M30 and M65) have been proposed as a possible indicator to perform a liver biopsy (60).

Chronic inflammation is the driving force for the onset and progression of fibrosis in NASH (61). Liver fibrosis represents, together with the comorbidities of metabolic syndrome, a significant prognostic determinant in NAFLD. For this reason, a major goal in NAFLD management is the prevention of fibrosis and its detection in the earliest stages to avoid progression to cirrhosis. Liver biopsy is the diagnostic gold standard for fibrosis. However, it is an invasive technique with possible complications, therefore non-invasive tests (NITs) have been identified (62). According to the latest EASL Clinical Practice Guidelines, non-invasive scores, serum markers, liver stiffness and imaging methods should be used for ruling out rather than diagnosing advanced fibrosis in low-prevalence populations and should be preferentially employed in patients at risk of advanced liver fibrosis (63). Crucial NITs in NAFLD patients stratification are especially the fibrosis-4 (FIB-4) - an index that takes into account age, transaminases and platelet count- and the liver stiffness evaluation by VCTE. As concerns fibrosis evaluation through cross-sectional imaging techniques, especially magnetic resonance elastography, their use is limited at the moment to tertiary referral centers and for experimental studies, in light of their cost, the limited availability and the procedural length. Finally, it is worth mentioning the new glutamate-serine-glycine (GSG) index which, combining three amino acids involved in glutathione synthesis, provides a good assessment of NAFLD severity and allows the discrimination of liver fibrosis (64).

## 4. The therapeutic role of GSH in NAFLD

As already mentioned, oxidative stress is a pathophysiological hall-mark of metabolic liver disease (65–68). Under this condition, ROS overproduction appears to be associated with an impairment of intracellular GSH homeostasis, leading to a reduction in GSH levels and in its antioxidant and hepato-protective function (69, 70). Based on these assumptions, a role for GSH in the treatment of liver disease has been hypothesized for NAFLD (71).

While several clinical studies examined the favorable effect of reduced GSH short-term or long-term administration on alcohol-induced liver diseases (72–76), the available literature on the effect of GSH on NAFLD is limited -see Table 1-, and the studies at hand are to be considered pilots (77–79).

**Table 1:** Main studies on the role of reduced glutathione (GSH) treatment in patients with metabolic liver disease.

Study	Country	Year	Type of the study	Study population	Treatment	Outcomes	Follow-up	Main findings	Ref
Dentico et al.	Italy	1995	50 pts 25 controls	NAFL No biopsies	1800 mg/day IV for 30 days (25 pts) 600 mg/day IM for 30 days (25 pts)	AST, ALT, GGT, bilirubin, MDA	4 month	↓AST, ALT and GGT ↓MDA No bilirubin improvement No adverse effects Efficacy high-dose treatment	77
Irie et al.	Japan	2016	15 pts	NAFL (n = 5) NASH (n = 10) Pre-treatment biopsies	300 mg/day PO for 3 months	ALT, GGT, 8-OHdG, IHC expression of GSH	3 month	↓ ALT, GGT and 8-OHdG in NASH (in NAFLD no statistical significance) GSH liver expression abundant (especially in NAFLD) Possible prevention of progression from NAFLD to NASH	78
Honda et al.	Japan	2017	29 pts	NAFL No biopsies	300 mg/day PO for 4 months	ALT, US (CAP)	4 month	↓ ALT CAP Improvement	79

ALT, alanine aminotransferase; AST, Aspartate aminotransferase; CAP, controlled attenuation parameter; GGT, gamma-glutamyl transferase; IHC, immunohistochemistry; IM, intramuscular; IV, intravenous; MDA, malondialdehyde; NAFL, non-alcoholic fatty liver; NASH, non-alcoholic steatohepatitis; PO, per os; pts patients; US, ultrasound; 8-OHdG, 8-hydroxy-2-deoxyguanosine; ↓, reduction.

An early work, presented in 1995 by Dentico and colleagues, evaluated the effect of 30-day administration of high doses of intravenous or intramuscular GSH on liver cytolysis indexes in patients with chronic steatosic liver disease (77). No adverse effects were reported and a significant reduction in liver tests (specifically transaminases and gamma-glutamyltranspeptidase -GGT-), with many cases of bio humoral parameters normalization, was detected in all treated patients, even several months after treatment interruption. In addition, confirming the efficacy of GSH treatment, a reduction in malondialdehyde, a marker of hepatic cell damage, was detected.

A subsequent study of Irie and colleagues in 2016 showed that the use of oral glutathione, at a daily dosage of 300 milligrams per day, may prevent NASH progression from NAFLD (78). A higher level of oxidative stress was detected in patients with NASH compared to NAFLD and a reduction in the levels of 8-hydroxy-2-deoxyguanosine (8-OHdG) and GGT, as markers of oxidative stress, was highlighted in NASH patients treated with GSH, with a consequent reduction of alanine transaminase (ALT). Also, they evaluated the immunohistochemical expression of GSH on pre-treatment biopsies, finding a stronger expression of GSH in NAFL than NASH. These results suggested a possible progression from NAFLD to NASH due to oxidative stress and demonstrated a potential therapeutic role for GSH in controlling the progression of liver damage.

The study by Honda et al., conducted in 2017, was an open-label, single-arm, multicentre pilot study that evaluated the therapeutic effect of oral glutathione administration (300 mg/day) in patients with NAFLD through the evolution of biochemical indices (ALT) and liver fat levels assessed by VCTE (79). ALT levels significantly decreased following treatment with GSH for 4 months, with a consequent decrease in liver fat levels non-invasively evaluated using elastography with controlled attenuation parameter.

These preliminary studies suggest a potential therapeutic effect of oral administration of GSH in NAFLD. However, the small sample-size, the short treatment period, the absence of control groups, the lack of liver biopsy evaluation after treatment are just some of the limitations of these studies. More studies are needed to elucidate the mechanism behind the effect of GSH and large-scale trials are necessary to confirm the therapeutic role for GSH. According to ClinicalTrials.gov, as of 5^th^ December 2022, no phase III clinical trial on the use of GSH in NAFLD is currently ongoing or recruiting.

## 5. Conclusion

NAFLD is a liver disease characterized by a high prevalence in the general population. Although several drugs are under investigation, there are currently no approved drugs for NAFLD (80). The complex pathophysiology and heterogeneity of the disease raises the speculation that combined treatment will be required for many patients. Therefore, the need for new therapies able to cure and prevent the progression of this condition is increasingly urgent.

The pathogenetic role of oxidative stress in NALFD is well known and would explain the rationale for the use of GSH as a potential therapy. The studies currently available on the use of both oral and parenteral GSH are promising but represent only pilot studies for the time being. Indeed, these studies are burdened by several limitations, most importantly the small sample size and the lack of evaluation of the therapeutic effect of GSH by liver biopsy, which to date is the gold standard for the definition of steatosis and fibrosis levels. Further studies are needed to confirm the actual benefit of this molecule on metabolic liver diseases and define the best route of administration and the most appropriate dosage, allowing its use in clinical practice.

## Author contributions

All authors listed have made a substantial, direct, and intellectual contribution to the work and approved it for publication.

## Funding

This paper was supported by “San Matteo Hospital Foundation, Internal Medicine research fundings, PRIN2017.”

## Conflict of interest

The authors declare that the research was conducted in the absence of any commercial or financial relationships that could be construed as a potential conflict of interest.

## Publisher’s note

All claims expressed in this article are solely those of the authors and do not necessarily represent those of their affiliated organizations, or those of the publisher, the editors and the reviewers. Any product that may be evaluated in this article, or claim that may be made by its manufacturer, is not guaranteed or endorsed by the publisher.
